# Health, Education, and Economic Well-Being in China: How Do Human Capital and Social Interaction Influence Economic Returns

**DOI:** 10.3390/bs13030209

**Published:** 2023-03-01

**Authors:** Tajwar Ali, Salim Khan

**Affiliations:** 1 Department of World History, School of History, Zhengzhou University, Zhengzhou 450001, China; 2 Business School, Zhengzhou University, Zhengzhou 450001, China

**Keywords:** Chinese General Social Survey, economic returns, employment, income premium, instrumental variable

## Abstract

In developing countries, it is generally believed that a good health status and education (human capital) bring economic well-being and benefits. Some researchers have found that there are overall financial returns and income premiums correlated with human capital because of its excellent and higher ability. Due to different views and a lack of consensus, the role of human capital is still ambiguous and poorly understood. This study investigates the economic returns of health status, education level, and social interaction, that is, whether and how human capital and social interaction affect employment and income premiums. Using the Chinese General Social Survey (CGSS) for specification bias, we used the instrumental variable (IV) approach to specify the endogeneity and interaction effect in order to identify the impact and economic returns of human capital and social interaction on the values of other control and observed variables. However, we show that an individual with strong and higher human capital positively affects economic returns, but the variability of these estimates differs across estimators. Being more socially interactive is regarded as a type of social interaction but as not human capital in the labor market; thus, the empirical findings of this study reflect social stability and that the economic well-being of socially active individuals is an advantaged situation. Furthermore, men with substantial human capital and social interaction are in a more advantaged position compared to women with similar abilities.

## 1. Introduction

**Figure 1:**
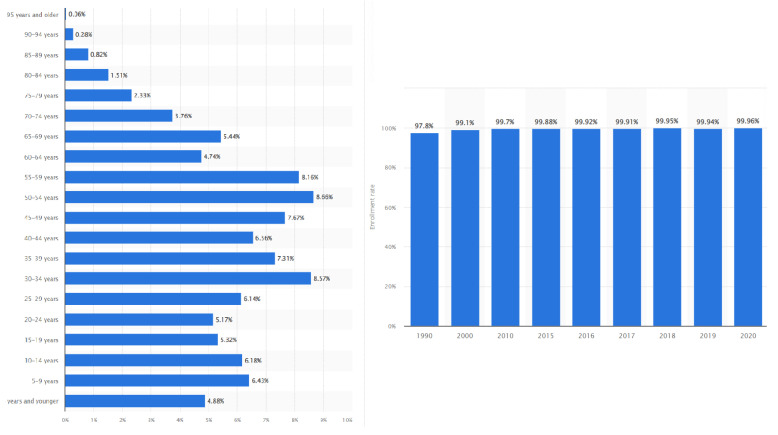
Population distribution and education level in China.

Since China’s continuous and swift social and economic development began over five decades ago with the land reform and others, inequality in human capital (education and health) and economic welfare have significantly attracted academic communities [1]. There is a consensus that inequality in both income and wealth has been rising in China and other emerging economies [2,3]. The previous studies on human capital and the social determinants of employment in China are wide-ranging [4,5,6,7]. However, with the vigorous development of the knowledge economy, since the 1990′s, education has become essential in promoting economic growth. As the highest level of education in the education system, the relationship between higher education and social and economic development is close. Higher education has become the engine of social, economic, and human capital development [8]. The contribution of human capital to economic development has become a significant factor in promoting actual sustainable development [9]. By the end of 2021, China’s population of over six-year-olds was 14,411 million, of which 99.96% had reached the primary education level (See., Figure 1). In other words, 99.96% of the population of China is formally literate, and between 2015 to 2021, the literacy rate of China grew by 0.106%, estimated by [10]. The State Council of China [11] stated that the enrollment rate of China’s higher education institutions reached 57.8% in 2021, compared with 30% in 2012. Among them, 6.7% have a college education level, 32.35% have a middle school education level, and 29.92% have a primary education level [12,13,14].

The number of graduates each year has also increased significantly with the expansion of universities. The Ministry of Chinese Education published a report on Educational Statistics [15] in 2020 that revealed that the number of graduates had reached 8.74 million. Such a huge group of graduates shows that 56% chose the postgraduate entrance examination, 5% chose civil servant roles, 5% chose to start their businesses and 30% chose employment directly, which amounted to 2 million graduates Even if they take postgraduate entrance examinations, they will still encounter employment problems after graduation.

Changes in the number and composition of the population in terms of education level and health status since the 5th census means that the human capital of the population in China has increased significantly due to the rapid development of the economy [16]. Concerning the number of people with various kinds of education per 100 thousand of the population, the number with a university education rose from 3611 in 2000 to 5098 in 2005, with an average annual increase of 7.14 percent. The number of people who reached the upper secondary education level increased from 11,146 in 2000 to 11,400, with an average annual increase of 0.45% [17]. China is facing an expansion of its urban population on a scale of exclusive growth that has not been seen in human history. The size of China’s population living in cities increased from 18% in 1978 to 56% in 2015 [18].

Human capital in developing countries is an essential social feature of the population [19,20,21,22]. Many theorists and researchers have tried to examine the individual’s economic return to education and health [23,24,25,26]. Many researchers have argued that individuals with higher education and good health could easily convert their human capital into a multidimensional economic return by acquiring high-profile jobs in the public sector [27]. Moreover, Munasib and Tian [28] have found that mentally and physically fit members of society are economically more vital because they obtain a higher income and employment premium in the labor market. The research findings of many social scientists suggest that acquiring political membership education status is the most important and influencing factor in obtaining a good job and a higher income in developing countries. Political membership in developing countries represents a unique social capital or social interaction because of a strong social network among party affiliates [29]. For instance, Gu and Zheng [30] showed that individuals with political membership, compared to those without membership, have approximately 10% more relatives or friends to help them find a high-profile job.

Similarly, other research studies have also revealed that individuals with higher human capital can quickly access more valuable socioeconomic and political information to acquire work-related benefits, including in regard to their choice of occupation or in social benefits such as documents and meetings [31,32]. According to previous research studies, human capital is the most essential and standard product reflected broadly in an individual’s earnings. Many research studies have stated that individuals with human capital and social interaction earn more income after controlling for an individual’s observed characteristics such as age, family background, locality, etc. [33,34,35]. Human capital brings significant socioeconomic returns, so the general public has a strong will to acquire a quality education and health care [36].

Consequently, individuals with high-quality education, good health, and strong political capital are likelier to get a comparatively more high-profile job with handsome pay in state-owned sectors, government enterprises, and state-run institutions. Sometimes in China, membership of the communist party is the main criterion for an applicant to be employed in the aforesaid influential units [37]; this is because political capital in China is reflected as a symbol of loyalty to the country. Many large private sector companies prefer individuals with political membership in China because their business strategy primarily aims to expand their good relations with the local administration. At the micro level, if an individual is employed in work, as mentioned earlier, their social and economic return is enhanced, possibly improving (their) personal well-being and comfort [38].

However, the empirical results of this research, as mentioned earlier, are mainly based on the Ordinary Least Square (OLS) method, which might be subject to bias. [19]. For example, the family’s higher ability or socioeconomic status might drive the empirical results of these OLS estimates, which would form a spurious and false association between education, health, and employment. Most research studies on the returns of human capital provide inadequate empirical evidence on individual unobserved characteristics. Therefore, this heterogeneity effect might confound and mix up any causal interpretation. Therefore, this paper recommends that the previous studies on the relationship between human capital and economic benefits are re-assessed. Thus, the current study analyzes the relationship between education, health, social interaction, and economic benefits by applying the binary logit model to quantify an individual’s employment stats. To solve the endogeneity problem, we applied the instrumental variable (IV) approach and interaction effect to test the impact of human capital and social interaction on economic returns and on the values of other independent variables (individual’s observed characteristics).

In the current study, we first tried to estimate the actual effect of an individual’s health status, education level, and social interaction on their economic benefits. Second, we tried to deliver a deepened level of knowledge about how the acquirement of human capital economically benefits an individual in China’s labor market. This study’s main novelty is that it considers the effect of omitted variable bias and estimates its actual return to human capital and social interaction. Therefore, to overcome the omitted variable bias, first, we controlled for several important factors that influence an individual’s economic return; afterward, we used another approach, named IV estimates.

The remaining parts of the article are ordered as follows: (II) the literature and hypothesis of the study, (III) data and their structure, variables, measurement of variables and descriptive statistics of the study, (IV) empirical strategy or econometric models of the study, (V) results and explanations of the proposed econometric models, and (VI) limitations, discussion and conclusion of the study.

## 2. Literature Review, Theoretical Framework, and Hypothesis

### 2.1. Literature Review

A consistent and higher level of human capital and social interaction holds great significance among policymakers; this is because education, health, and social interaction are the leading indicators of human capital and social interaction. Mainly, the linkages between education and economic return (in the form of employment and income) have been empirically analyzed by many scholars. Many researchers have emphasized that education is the leading indicator of human capital, considering health status [39]. Some studies emphasize that growing economic returns to education can explain the main part of human capital. Hannum, Zhang [40] shows that the returns to education for women are higher than for men because most women trade their income for their husband’s income. Psacharopoulos and Patrinos* [41] reveal that returns to education are approximately, on average, 10.6%; meanwhile, this rate in developing countries is in the range of 12%, and the economic returns to education in developing countries is 10%.

Furthermore, many researchers have studied the relationship between health status and employment level, while most empirical examinations of health and economic returns are based on developed economies. Andreeva, Brenner [42] suggest that poor health status (in the form of obesity) is the main factor that withdraws people from the labor force into unemployment. Similarly, the work of Yahong and Khan [43] analyzed the cross-sectional investigation of education, health, and employment level. The empirical findings of the econometric model suggest that people with higher education are more likely to get better jobs. Likewise, healthy individuals were more likely to be employed than relatively less healthy individuals. The empirical result opposed the multiplicative effect of gender inequality.

From a methodological point of view, several past research studies have used the well-known OLS approach to analyze the effect of human capital on an individual’s income and economic return. At the same time, they have found positive returns for individuals with higher education compared to an individual with a lower level of education. Meanwhile, an individual’s human capital, such as education and health status, might be associated with characteristics not observed in the model—for example, the family background of respondents and an individual’s abilities. However, the estimation of the OLS model might be subject to bias (omitted variable bias) even though a few past research studies have tried to address the endogeneity problem by using the panel data approach. For instance, some studies have used the instrumental variable (IV) approach, the within-twin fixed effect (FE), and the Heckman selection model [44,45,46], while the others overlooked the problem of endogeneity [47,48]. Ma and Iwasaki [49] utilized a unique estimating method named the recalled panel data approach; by using this approach, they pooled cross-sectional data, including data gathered by asking questions based on a survey. They asked respondents about their level of education and health, and their economic status (income). The main problem with this method was an error in the measurement because it is usually hard for respondents to remember many things, such as when they got their job, when they last went to the hospital or when their income had increased in the previous few years. However, Gore [50] and Markussen and Ngo [27] used the panel data approach and showed positive returns for individuals with social interaction. However, they did not provide sufficient evidence regarding variation, that is, how much variation he observed in his study in the generally time-invariant system.

In the case of Vietnam, Vu [51] used parental education and occupation as the instrumental variables, and he showed that the OLS estimates are smaller than the IV estimates. Similarly, Soumerai and Koppel [52] and Ivlevs and Hinks [53] used parental education and occupation as instruments. However, we criticize this approach and claim that the exclusion of the father and mothers’ occupations and level of education might not result in valid instruments because these factors may be associated with families’ socioeconomic characteristics and, therefore, might positively influence income. Thus, parental occupation and education should be used as essential determinants of individual earnings and be included in the control group. On the one hand, Li, Liu [23] used an estimator called the within-twin fixed effect to control directly for characteristics that are not observed. Based on their empirical analysis, they could not show enough evidence regarding the positive impact of middle education on an individual’s income. The main inspiration for the use of the within-twin fixed effect was that it controls for the omitted variable’s time-invariant system as they have the same family backgrounds and natively similar abilities. Li, Liu [23] found insignificant and minor results for an individual with political membership, and also determined that the direct and significant results of the OLS approach show advantageous family backgrounds and a higher personal ability, rather than a causal effect of the membership.

On the other hand, a few studies, such as by Bound and Solon [54] and Neumark [55], have claimed that the twin FE approach could not remove the characteristics that are not observed. In other words, the within-twins FE estimator does not solve the problem of bias due to endogeneity; instead, it heightened it. By that, we mean that the differences amongst twins cannot wholly overcome the omitted variable bias problem since monozygotic twins are identically different. To be precise, twins that are unrelated genetically are not the same in their characteristics, for example, in their personal abilities, temper, and spirit of doing something. Therefore, we concluded that this approach heightened the problem of error in measurement, and that it still technically and theoretically faces the problem of omitted variable bias.

### 2.2. Theoretical Framework

Dual market or segmented economy theories originated from challenges in interpreting persistent structural inequalities, despite increases in invisible capital (social or human). It would seem that the structural economic idea of economic returns determination, including dual market or segmented theories, could help understand some of the flaws that are evident in the modern economic structure [56,57]. Nemours’ theories related to dualism and segmentation argue that the identification of the restructuring of the best-fit job is a source of both the skilling and deskilling of workers across the economy [58].

Considering the inconsistent conclusions in regard to the impact of human capital and social interaction on economic returns, scholars have confirmed that human capital and social interaction alone are deficient as explanations of inequality in employment opportunities and other economic returns (between the different classes) [59]. Some empirical analyses suggest that gender and race determine employment opportunities beyond the effect of education and health. In other words, race and gender can further divide the market structure via the labor force within employment opportunities [60]. Frederiksen [61] examined the employment separation procedure to study gender inequality in employment and job constancy. As a result of the investigation of this phenomenon, a few points were found; First, that women have a high unconditional probability of being separated from employment; second, that there is no gender inequality in the labor market; and third, that women’s employment stability is relative to men. Similarly, Yabiku and Schlabach [62] examined the effect of human capital (in terms of education level) on employment opportunities. Using the Discrete-Time Event History Model (DTEH), they found that school attainment upsurges the probability of employment, while school enrollment tends to delay it.

### 2.3. Hypothesis

Based on the literature review and the theoretical framework, this study hypothesized that individuals with higher education, a good health condition, and other social interaction could enhance their multidimensional economic returns. This included, for instance, the probabilities of being employed, being a workplace manager, and increasing one’s income level. Therefore, the most critical and new sub-point is the gender gap in the economic returns of people with similar human and social interactions. Thus, we have proposed the following research hypotheses.

**Hypothesis** **1-H1.** *education, health, and social interaction positively affect employment probabilities and income. Remarkably, men can improve their economic return more than that of women.*

**Hypothesis** **2-H2.** *an individual’s education, good health, and income has a significant positive effect, and the father’s education has a negative or even null impact on an individual’s observed characteristics.*

## 3. Materials and Methods

### 3.1. Data

The main aim of the current study was to expand the overall understating of the economic return of individuals with higher human and social interaction. This paper mainly examines the effect of health status, education, and social interaction on financial returns. For the empirical analysis of this study, we used Chinese General Social Survey (CGSS) data, which are nationally representative multi-stage stratified sampling data. These stratified sampling data are obtained via a yearly survey of China’s both rural and urban households, and collect data on socioeconomic tendencies and fluctuations in the relationship between the quality of life and China’s socioeconomic structure (Wang et al., 2019). As stated in the CGSS report, it mainly comprises two survey phases from 2003 to 2019. For this empirical analysis, we utilized the survey data of CGSS from the second phase because these data track well-being very closely.

The CGSS data survey implemented a proportionate and multi-stage stratified sampling technique. As primary sampling units, the CGSS gathered data from 100 districts (counties) and 5 big cities, including Shanghai, Guangzhou, Beijing, Shenzhen, and Tianjin, in the first step. In the second sampling step, 4 village committees or neighborhoods were randomly selected in every district where the sampled respondents were selected. Similarly, in the last step, approximately 25 sampled households were selected from each village committee or neighborhood, while 1 individual from every household unit was selected for interviews randomly. In the abovementioned 5 populated cities, 80 committees were established. Similarly, in this way, approximately 480 neighborhood committees were interviewed countrywide. The answering rate of the sampled respondents in this survey was more than 70%.

This survey was perfect for the empirical analysis of this study as it gathered general facts and figures on the subject in relation to the sampled respondents’ employment status, level of income, education, health status, social interaction, and other socioeconomic characteristics. The second phase of the CGSS data survey is accessible openly (from 2010 to 2019). To create a sizeable analytical sample set, we employed the joined sampled survey sets of CGSS 2013 and CGSS 2015 for the analysis of this study.

### 3.2. Variables’ Descriptions and Measurement

We used the multiple-stage questions employed by Sato and Eto [20], including (“are you employed?”, “are you a senior or middle manager at your workplace” and “what is your income”) to assess an individual’s employment status. In the CGSS dataset, the options available to the respondent were as follows: “employed” or “unemployed”, and “manager” or “not”; meanwhile, both full-time employed and part-time employed individuals were counted up as being employed and coded as 1, and those unemployed or otherwise were coded as 0. Similarly, to reduce the dependence on only one single item measure (employment), we created a substitute variable measure; this was named manager position at the workplace. In other words, this study systematically used the additional dependent variable to replicate an individual’s economic return in China. Given the available data, we evaluated the manager position at the workplace through the two items “I am a manager” (middle or senior manager) and “I am not a manager”; the sampled respondent had to choose one of the two abovementioned options. We coded 1 for the old or middle manager at the workplace; otherwise, we coded 0. Afterward, we used the log form of the annual earnings to capture the net income status of the sampled respondent.

The primary predictor variables for human capital (individual’s health status, education level, and social interaction) were derived from the proposed question about an individual’s participation. Respondents had to choose only one of the available options, for example, 1 = unhealthy status, 2 = general health status, 3 = relatively healthy, and 4 = very healthy. Similar options were also available for levels of education, health status, and social interaction. In the combined data set of CGSS 2013 and CGSS 2015, there were 10,969 sampled observations after losing the missing value. To reduce the bias caused by the omitted variables, we controlled for other important individual-level characteristics. Some of these controlled group individual-level characteristics were controlled for the first time; this group of variables did not simply emphasize factors that were related to individuals, but also emphasized variables that were related to family background. This group of variables was associated with an individual’s gender, area of residence, religion, age, ethnicity group, etc. Other group-controlled characteristics that were linked to an individual’s family background included the father’s education, mother’s education, and parental employment status, etc.; we also used yearly and provincial fixes to control for the heterogeneity in the model estimation. The detailed definitions and descriptions of the abovementioned variables for our econometric analysis are listed in Table 1.

### 3.3. Descriptive Statistic

In the CGSS 2013 and CGSS 2013, urban and rural, approximately 11.9% of the whole sample was found to be an active population. Table 2 shows general variations between respondents with active and non-active members of the society based on our calculation. As per the figures Table 2 reports, there were sizable dissimilarities between the active and non-active population. On average, compared to non-active individuals, active individuals were male, earned more, were employed more, and had a managerial position at the workplace. Additionally, more than 50% of the respondents with an active status had passed senior secondary school, college or equivalent education.

In comparison, only 29% of individuals with a non-active status had achieved the corresponding education levels. There was a considerable difference between the two groups’ job positions and areas of residence. Many respondents with an active status lived in urban areas and work as middle managers or senior managers at workplaces. On the other hand, the majority of individuals with non-active status were engaged in ordinary working (agriculture, skilled, unskilled, etc.) positions at working units.

### 3.4. Empirical Strategies

In the first part of the empirical strategy, we used the binary logistic regression model to examine whether an individual’s health, education, and social interaction affected their employment status (Normal employment/manager position at the workplace). We tested the proposed Hypothesis 1.

We estimated the following regression model:

*P(Y) = 1/1 + e^−(*β*_0_ + *β*X_i_)^*

To express the dichotomous dependent variable, the study used the logit model. It assumes the above cumulative probability function, where *P*(*Y*) = the probability that the individual is employed; *e* = the exponential value; β = the row vector of the parameters; and *X_i_* = the column vector of the variables. Meanwhile, *P*(*Y*) symbolizes the probability of occurrence that cannot be observed directly. Therefore, (0, 1) as a binary variable was developed, in which the value of “1” represents an individual who is employed and “0” represents an individual who is not. For this, we can derive a regression equation directly from the above logistic probability density function:

$$P\left(Y\right)=\frac{1}{1+e^{-yi}}=\frac{e^{yi}}{1+e^{yi}}$$

where *Y* is nothing but β_0_ + β*X_i_*; this function can be further expanded by adding variables such as the following:

$$P\left(Y\right)=\frac{1}{1+e^{-\left(b1+b2Edui+b3Healthi+b4Soci+Xiµ\right)}}\;$$

$$P\left(Y\right)=\frac{e^{\left(b1+b2Edui+b3Healthi+b4Soci+Xiµ\right)}}{e^{\left(b1+b2Edui+b3Healthi+b4Soci+Xiµ\right)}+1}$$

where *Edu* is the level of education (*Edu*1, *Edu*2, *Edu*3, and *Edu*4) and health is an indicator of an individual’s health status (such as unhealthy, generally healthy, relatively healthy, and very healthy). Similarly, Soc is an indicator of an individual’s social interaction (rarely, sometimes, and very often). *X* is a vector of individual other characteristics (called control group) comprising area of residence, gender, age, etc. In addition, “*P*(*Y*)” is the chance of occurrence or the probability that an individual is employed. Considering this, the probabilities of occurrence of an individual who is not employed or unemployed will be as follows:

$$1-P\left(Y\right)=\frac{1}{1+e^{yi}}\;$$

Therefore, this can also be rewritten as follows: $\frac{P\left(Y\right)}{1-P\left(Y\right)}$ = $\frac{1+e^{yi}}{1+e^{-yi}}$ = eyi

Hence, $\frac{\left(\frac{P\left(Y\right)}{1-P\left(Y\right)}\right)i\;}{\left(\frac{P\left(Y\right)}{1-P\left(Y\right)}\right)j}$ = $\left(\frac{Odds\;of\;employment\;event\;“i”}{Odds\;of\;employment\;of\;event\;“j”}\right)$ is the odds ratio of employment.

In the second part of the empirical strategy, we further observed a much closer relationship between the individual’s earnings and their health, education, and social interaction. Given that the other indicators and demographic variables were the same, we implemented the human capital earning equation function introduced by Mincer (1974), with an indicator for our leading indicator as an elementary background. The functional specification of the model is as follows:

*log (income_i_) = *β*_*1*_ + *β*_*2*_Edu + *β*_*4*_Health + *β*_*3*_Soc + X_i_µ + Ɛ*

where *income_i_* represents an individual’s income; according to the rule of thumb, the resulting coefficient of β’s will not be unbiased if the other unobserved variables are in the model’s error term (Ɛ). This affects the independent variable (health, education, social interactions, and other control variables) and the dependent variable (income). If the unobserved variables are apprehended in ε, the OLS’s estimated results would probably be biased due to the direct association with human capital and income in the above equation. To address our model’s possible endogeneity, we used a corresponding method called the instrumental variable(s) (IV) model. For the causation of unobserved variables, we used the father’s education as an instrument for human capital.

Meanwhile, as we know, our main variables are binary. Therefore, we used (Wooldridge, 2002) two-step instrumental variables approach to consider the binary nature of the endogenous variable. We analyzed a logit model for human capital and proceeded with the fitted resulting probability values. After that, we used the achieved probabilities fitted values as an instrumental variable for individuals who achieve party membership (see above equation). The two-stage instrumental variable approach’s main advantages are, firstly, its robustness to misspecification because we used the fitted value of probabilities as an IV for an individual’s human capital. Secondly, this approach is more well-organized and effective than the 2-SLS approach because, in the logit model, there is a 0th (zeroth) stage of constraints in which the probabilities of the fitted values have to occur between one and zero. In addition, it eliminates the nonsense deviation in the fitted value of possibilities that is achieved from the linear model, specifically the probabilities outside the range of one and zero.

Contrary to the significance assumption, it is well known that elimination constraint assumptions cannot be tested. However, in order to see the empirical evidence on the association between a father’s education and an individual’s observed leading characteristics, we tried to support the credibility of the instrument variable. During this process, a relationship between these measures was not found, and it was clear that the instrument(s) could not empirically demonstrate that the it could satisfy the restriction’s exclusion due to the fact that an association with an undetected capacity might still exist. However, to address the instrument’s reliability, it is helpful if the father’s education is not a key factor of quantifying an individual’s selected characteristics once the mother’s parental level of education is controlled. We can also clarify that the characteristics already observed between individuals whose fathers have higher education (treatment group) and those whose fathers have education (control group) must not be different. We have tested this issue on numerous individual characteristics while focusing on observables significantly and highly associated with ability. More specifically, we focused on the overall testing procedures that we performed in order to inspect the endogeneity of the father’s education. Finally, we ran the respondent’s measures on the father’s education, health, social interaction, and other control variables that we included in our central description and revealed that the father’s education level was not correlated with characteristics that belong to the individual. Therefore, we presented three more hypotheses from the discussion mentioned above.

## 4. Results and Discussion

The binary logistic regression results are reported in Table 3, which reports an individual’s probability of being employed. Model 1 to Model 4 correspond to the controls for gender, indicators of human capital, and other controls such as age, area of residence, religion, ethnicity, etc. In terms of employment differences across gender, Model 1 of Table 3 reports that after controlling for age, area of residence, religion, ethnicity, provincial fixed effect, regional fixed effect, and yearly fixed effect, men’s probability of being employed are 0.273 (e ^(0.242−1)^) higher than women. After controlling the individual’s education, Model 2 shows that the difference between an individual’s probabilities of being employed across gender was statistically positive but that it declined.

This indicates that the gender difference in achieving employment opportunities still exists, and this difference is mainly due to differences in the many social and cultural hurdles. However, the results of Model 2, Model 3, and Model 4 constantly show that an individual’s education level, health status, and social interaction significantly and positively affect the probability of being employed.

The resulting probabilities of Model 4 (full model) of Table 3 show that the coefficients of Ghlth, Rhlth, and Vhlth (Ghlth = good health status; Rhlth = relatively good health status; Vhlth = very good health status) were estimated to have 0.19 (0.181), 0.22 (0.201), and 0.33(0.288) probability values, respectively. All levels of health status were statistically significant and positively affected the employment probability. Similarly, education status and social interaction were statistically significant and different from zero. Individuals with higher education (such as above middle school, etc.) and more social interaction (more friends, more relatives, and more relationships with people) have more chances of obtaining employment. Therefore, we observed a positive effect of human capital and social interaction on an individual’s probability of being employed, but gender differences and the disadvantageous position of women still exist. The empirical findings obtained here are in line with the empirical investigation of Blackburn, Jarman [63], and Klasen and Lamanna [64].

The alternative logistic regression model of “manager position at the workplace” in Table 4 shows the findings of the same results where we used manager position at the workplace as a dependent variable (coded as, 1 otherwise as 0). Model 4 (full model) of Table 4 shows that men were more likely to become workplace managers. Likewise, individuals with good health conditions were more likely to become a manager in the workplace. This phenomenon indicates that men, especially mentally and physically healthy men, have leadership qualities in economic activities compared to women. The study’s findings are consistent with Stamarski and Son Hing’s [60] results for gender discrimination models. Thus, overall, Hypothesis 1 is confirmed: 

### 4.1. Explanation of the Positive Effect of Human Capital on Employment

From the descriptive statistic, we inferred that most healthy, educated, and socially interactive individuals (men–women) were more likely to live in urban areas, where employment opportunities and market activities were sufficient. The direct significances are individuals (women or men) with higher socioeconomic power in families living in urban societies. Additionally, higher education makes it easy to find a good job (such as a senior or middle manager’s position at the workplace) and participate in market activities. These facts bring about higher socioeconomic and political power among urban neighborhood residents. With more substantial socioeconomic influence, women had to reject society’s outmoded arrangement in which women have to work inside the home and men perform agricultural activities. Thus, an individual’s employment status depends on their human and social interaction, whether they are well educated and participate in social activities.

We start the estimation process by utilizing the proposed OLS model to analyze the income gap between active and non-active individuals. Nevertheless, the resulting coefficients of these analyses might not be clarified as essential because these estimations do not consider the problem of endogeneity in models as a whole. Model 1 of Table 4 shows the significant differences in the resulting coefficients across gender without a control for health, education, and social interaction (we add a limited power, such as the area of residence, age, religion, etc.). It shows that men earn 45% (*e* ^(0.372−1 × 100)^) more than women before controlling for the individual’s characteristics. Meanwhile, after controlling for health status, Model 2 of Table 5 shows that the income gap between men and women statistically decreased from 45% to only 24% (*e* ^(0.220−1 × 100)^). This shows that if the individual is healthy in a working population, the individual’s income will be considerably increased. The empirical findings obtained here are in line with the work of Ma and Iwasaki [49] for the meta-analysis, Ma [65] for the Chinese labor market, and Tian and Zhang [66] for China.

Additionally, the income gap between men and women in Chinese society was mainly due to differences in the acquirement of education [67]. In model (3), we did not report the coefficient values of the levels of education so that we could avoid overlapping in the table. However, all of the resulting coefficients were statistically significant and different from zero. After adding the education levels, the gender gap dropped considerably.

In contrast, after adding the social interaction and education in Model 3 and Model 4 (full model), respectively, to some extent, it reduced the resulting coefficient of education very slightly. Although all the models included provincial, regional, and yearly fixed effects to overcome the biasness and weak estimation. In addition, the four models’ results constantly show that health status, education level, and other individual characteristics have a positive and significant effect on an individual’s overall income and an inverse effect on the gender gap. Therefore, the proposed Hypothesis 2 is satisfied and confirmed. These findings are contrary to the work of Liu and Liu [68] for the Chinese economy.

### 4.2. Effect of Father’s Education on Individual’s Health Status

The estimation of the multiple regression model does not consider characteristics that might be associated with the individual’s human capital and social interaction. The analysis of the OLS provided preliminary and initial empirical evidence for understating the actual effect of human capital and social interaction on income. However, the OLS approach cannot capture the inherent problem of endogeneity. The well-known assumption that all the variables are unbiased due to endogeneity may not be realistic either. Therefore, we used the father’s education as an instrumental variable to report the most probable endogeneity due to these individual-level characteristics. Before addressing the estimation of IV, this part of the study deliberates on the actual effect of IV on the resulting probability of human capital. In addition, we estimated the reduced form model to ensure that the proposed instrument was sufficient and robust.

Table 6 presents the resulting probabilities when we started the analysis of the human capital in models (1), (2), (3), and (4) from the binary logistic regression model. All models consistently show that the father’s education influences an individual’s probability of accumulating higher human capital and reducing the gender gap. The estimated coefficients in Model 4 of Table 6 show the effect (marginal effect) measured at the mean value as a whole. The impact of the father’s education on an individual’s human capital was positive and highly significant (with a common coefficient sign of 0.095). At the same time, the estimation of the models was robust: whether to include other individual-level characteristics and provincial or regional fixed effects.

Furthermore, models (1), (2), (3), and (4) of Table 7 reveal the direct relationship between an individual’s income and their father’s education. As long as the father’s education enhances the individual’s education probability, the relationship between the father’s education and an individual’s income level should emerge in the reduced-form model. We were not surprised because we found the expected coefficient pattern that fully emerged in Table 7 (full model). Therefore, we observed that a father’s education had a highly significant and direct effect on an individual’s education. That is, an educated father was a critical predictor of an individual’s education. We also found a slight but significant and direct relationship between the father’s education and the individual’s income, as shown in Table 7. Hence, overall, Hypothesis 2 is confirmed again.

### 4.3. Interaction Effect

Next, we report the estimates of the interaction terms in our regression models. In Table 8, we use a single equation to check the two-way interaction effect between health status (very healthy), gender, education, and social interaction with the main effect. In Model 1 (income model) and Model 2 (employment model) of Table 8, we interact very healthy health conditions (Vhlth) with gender. The education level of individuals interacted with hlth3 in Models 3 and 4, and health status and social interaction in Models 5 and 6 in Table 8.

The interaction between Vhlth and gender (male and female) is statistically significant, with negative results in both Models 1 and 2. The negative and statistically substantial results from the coefficients of the interactive term (“hlth3*gender”) imply that men benefitted more in income and employment. Similarly, all of the two-way interactions between Vhlth3 and an individual’s level of education, and their effect on income, are statistically significant even at a 1% level in Model 3 of Table 8. Still, none of the interactive terms are statistically substantial, except for the interaction between Vhlth*Edu2 (Vhlth and senior secondary school education) in Model 4. As shown by the interaction effect analysis, we also observed that an unexplained income gap besides the effect of Vhlth, such as gender, education, and social interaction, remains an important issue in China’s labor market. Table 8 proves that gender, education level, and social interaction still influence an individual’s income. The analysis of interaction terms revealed some important points; for example, man has a positive effect on both income and employment models. However, in the employment model (Model 2), the two-way interaction effect is stronger than the interaction effect of the income model. Education (with higher degree and Vhlth) also significantly increases an individual’s income relative to being only educated. The interaction effect was strongly significant for the income model but significant for the employment model (Model 4). The findings of the main effect were consistently statistically significant and positive in Model 5 and Model 6, but the coefficients of the interaction terms did not achieve statistical significance. This finding can be justified by the work of Liu, Xing [69] for the Chinese economy.

## 5. Conclusions

To evaluate the actual specification bias and interaction effect of human capital and social interaction on an individual’s economic return, we observed and controlled for as many essential income and employment influencing factors as possible. However, there may still be some omitted variables because of the unavailability of data in the CGSS 2013 and 2015 datasets. One of these variables might be the personality of an individual. According to Lyubomirsky, Tkach [70], and Cheon and Lim [71], personality traits explain more than 45% of the deviation in the well-being of an individual. They also argued that an individual’s disposition and personality traits are more associated with an individual’s well-being compared with life events, demographic and circumstantial factors. This is an obvious and very critical limitation of this study. Additionally, our study was based on an individual’s recorded employment and reported income (open economy activities), and does not consider an individual’s unrecorded income and employment (underground economy activities). For example, if a healthy, educated, and socially active person is associated with the underground economy’s activities, our estimates overestimate the economic return of human capital and social interaction.

In the present study, we empirically investigated the impact of human capital (health status and education level), along with social interaction, on economic return (employment and income). In previous sections, we hypothesized that individuals with substantial human capital and social interaction were more likely to attain high-profile jobs, higher incomes, overall other economic returns, and socioeconomic status. Our empirical results show that individuals with strong human capital can obtain higher economic well-being. The main focus of our study was to examine the empirical evidence on economic returns to human capital, while investigating the interaction effect and specification bias by using the instrumental variables and the method of interaction effect in regression models. The findings of previous studies show that employment and income premium are correlated with higher and strong human capital because of their higher level of ability [72]. However, our resulting coefficients suggest a sizeable and robust economic benefit to human and social interaction, even after controlling for other observed characteristics. In the overall analysis, we revealed that men with strong human capital are more likely to enjoy a high-profile job and a higher income. Our analysis shows that men with higher education and a good health status in China benefit more than women. Overall, based on the analysis of this study, this research primarily induced some critical points. Firstly, obtaining higher education does not pay equally across genders, indicating women’s disadvantageous position and the gender bias of economic and social activities. Secondly, an individual’s ability to acquire higher education, health facilities, and earnings remains dependent on their fathers’ education to some extent. Thirdly, the dependency of the other characteristics of an individual is not dependent on their fathers’ education.

### Limitation

The current study’s findings are essential to generalizing this research area to other emerging economies like China, but not without specific limitations that should be considered in future studies. This study used health status and education level as crucial human capital indicators. To extend the current analysis in an auspicious way, it would be better to use other indicators of human capital and compare whether the findings of this study are consistent and robust. Furthermore, we examined the impact of human capital on economic return by incorporating social interaction as an essential control variable in the Chinese economy. Therefore, we demonstrate a research gap for future researchers to investigate a similar issue using another reliable indicator: social capital. In addition, we offer an empty gap for future research to examine the impact of human capital on economic returns in other emerging or under-developed economies, such as Sub-Saharan African countries.

## Figures and Tables

**Table 1:** Description and definition of variables.

Variables	Description and Definition	
Emp	The employment level of individual	1 = if the individual is employed 0 = otherwise
lninc	Income	Log form of an individual’s yearly income (RMB)
Gender	Gender of individual	1 = male 0 = otherwise
Edu	Education level of individual	0 = junior secondary school education or below 1 = senior secondary school education or equivalent of it 2 = college diplomas or equal 3 = bachelor’s degree or above education
Health_st	Health status of individual	1 = unhealthy status 2 = general health status 3 = relatively healthy 4 = very healthy
Social_int	Social interaction	1 = sometimes 2 = mostly
F_Edu	Father’s level of education	The exact definition as abovementioned (0–4)
M_Edu	Mother’s levels of education	The exact definition as abovementioned (0–4)
Area	Area of residence	1 = urban 0 = rural
Rel	Religion	1 = believers 0 = otherwise
Age	Age of respondent	Individual’s age in years (continuous variable)

Data source: CGSS 2013 and 2015.

**Table 2:** General characteristics of individuals with active and non-active status.

Variables	CGSS 2013–2015	
	Active	Non-Active
Employment	0.835 (0.587)	0.598 (0.410)
Manager position at the workplace	0.533 (0.510)	0.257 (0.434)
Log form of yearly income (RMB)	10.673 (3.944)	10.292 (3.423)
Male	0.703(0.490)	0.505 (0.493)
Education		
junior secondary school education or below	0.351 (0.441)	0.623 (0.489)
senior secondary school education or equivalent	0.332 (0.470)	0.242 (0.321)
college diploma or equivalent education level	0.250 (0.319)	0.121 (0.293)
University-level degree or above education	0.066 (0.100)	0.003 (0.050)
Health		
Unhealthy	0.121 (0.302)	0.127 (0.295)
General healthy	0.489 (0.497)	0.501 (0.476)
Relatively healthy	0.285 (0.473)	0.261 (0.422)
Very healthy	0.105 (0.301)	0.110 (0.309)
Social interaction		
Sometimes	0.460 (0.401)	0.415 (0.392)
Mostly	0.387 (0.398)	0.303 (0.323_
Urban	0.692 (0.510)	0.512 (0.487)
Religious (believers)	0.095 (0.121)	0.157 (0.225)
Age	54.511 (15.16)	51.934 (14.20)
Total observations	1306	9663

Note: we used the Chinese General Social Survey (CGSS 2013 and 2015) data for urban and rural areas. The above-displayed variables are used in the current study. Due to a missing value, the calculated percentage of manager positions is based on 5010 observations, while the standard deviation is stated in the parenthesis.

**Table 3:** Logit regression on employment return to human capital, CGSS 2013 and 2015.

Dependent: Employment				
	Model 1	Model 2	Model 3	Model 4
Female ^b^ Male	0.242 *** (0.079)	0.231 *** (0.090)	0.226 *** (0.087)	0.220 *** (0.079)
Hlth1 ^b^ Ghlth		0.191 *** (0.041)	0.181 *** (0.061)	0.984 *** (0.072)
Hlth1 ^b^ Rhlth		0.299 ** (0.077)	0.201 *** (0.069)	0.156 *** (0.070)
Hlth1 ^b^ Vhlth		0.483 *** (0.011)	0.381 *** (0.071)	0.286 *** (0.081)
Edu1 ^b^ Edu2			0.152 *** (0.061)	0.181 *** (0.045)
Edu1 ^b^ Edu3			0.296 *** (0.084)	0.201 *** (0.080)
Edu1 ^b^ Edu4			0.300 *** (0.078)	0.288 *** (0.071)
Social interaction				0.095 ** (0.041)
Control Group	Yes	Yes	Yes	Yes
Provincial effect	Yes	Yes	Yes	Yes
Year effect	Yes	Yes	Yes	Yes
Regional effect	Yes	Yes	Yes	Yes
Pseudo R^2^	0.1613	0.1963	0.2101	0.2210
N	10,969	10,969	10,969	10,969

Robust standard errors are presented in parentheses. *** denotes that the resulting coefficient is significant at the 1% level. ** denotes that the resulting coefficient is significant at a 5% level. ^b^ indicates the reference category.

**Table 4:** Logit regression on manager position at workplace, CGSS 2013 and 2015.

Dependent: Manager Position at the Workplace				
	Model 1	Model 2	Model 3	Model 4
Female ^b^ Male	0.223 *** (0.080)	0.203 *** (0.055)	0.188 *** (0.63)	0.186 *** (0.58)
Hlth1 ^b^ Ghlth		0.051 ** (0.012)	0.057 *** (0.016)	0.062 ** (0.022)
Hlth1 ^b^ Rhlth		0.155 *** (0.062)	0.113 ** (0.065)	0.111 * (0.080)
Hlth1 ^b^ Vhlth		0.232 *** (0.094)	0.212 *** (0.089)	0.207 *** (0.075)
Education	No	No	Positive	Positive
Social interaction	No	No	No	Positive
Control Group	Yes	Yes	Yes	Yes
Provincial effect	Yes	Yes	Yes	Yes
Year effect	Yes	Yes	Yes	Yes
Regional effect	Yes	Yes	Yes	Yes
Pseudo R^2^	0.1113	0.1363	0.1901	0.2010
N	5010	5010	5010	5010

Robust standard errors are presented in parentheses. *** denotes that the resulting coefficient is significant at the 1% level. ** denotes that the resulting coefficient is significant at a 5% level. * Denotes that the resulting coefficient is significant at a 10% level. ^b^ indicates the reference category.

**Table 5:** OLS Models on the return of income to human capital.

Dependent: log Income				
Varibales	Model 2	Model 3	Model 4	Model 5
Female ^b^ Male	0.372 *** (0.049)	0.220 *** (0.055)	0.199 *** (0.039)	0.192 *** (0.044)
Hlth1 ^b^ Ghlth		0.110 *** (0.021)	0.107 *** (0.018)	0.094 *** (0.019)
Hlth1 ^b^ Rhlth		0.134 *** (0.033)	0.135 *** (0.038)	0.129 *** (0.040)
Hlth1 ^b^ Vhlth		0.198 *** (0.039)	0.202 *** (0.043)	0.210 *** (0.038)
Education	No	No	Positive	Positive
Social interaction	No	No	No	Positive
Control Group	Yes	Yes	Yes	Yes
Provincial effect	Yes	Yes	Yes	Yes
Year effect	Yes	Yes	Yes	Yes
Regional effect	Yes	Yes	Yes	Yes
R^2^	0.1311	0.2027	0.2101	0.2311
N	10,969	10,969	10,969	10,969

Robust standard errors are presented in parentheses. *** denotes that the resulting coefficient is significant at the 1% level. ^b^ indicates the reference category.

**Table 6:** Logit model on the relationship between father’s education and individual’s education, CGSS 2013 and 2015.

Dependent: Human Capital (Education)				
	Model 2	Model 3	Model 4	Model 5
Female ^b^ Male	0.192 *** (0.027)	0.141 *** (0.033)	0.134 *** (0.041)	0.128 *** (0.39)
FEdu1 ^b^ FEdu2		0.045 *** (0.012)	0.032 *** (0.016)	0.029 *** (0.022)
FEdu1 ^b^ FEdu3		0.107 *** (0.022)	0.110 *** (0.034)	0.108 *** (0.041)
FEdu1 ^b^ FEdu4		0.140 *** (0.034)	0.141 *** (0.029)	0.143 *** (0.031)
Health status	No	No	Positive	Positive
Social interaction	No	No	No	Posotive
Control Group	Yes	Yes	Yes	Yes
Provincial effect	Yes	Yes	Yes	Yes
Year effect	Yes	Yes	Yes	Yes
Regional effect	Yes	Yes	Yes	Yes
Pseudo R^2^	0.0711	0.0927	0.0981	0.0991
N	10,969	10,969	10,969	10,969

Robust standard errors are presented in parentheses. *** denotes that the resulting coefficient is significant at the 1% level. ^b^ indicates the reference category.

**Table 7:** Reduced form estimates on the relationship between father’s education and individual’s earnings, CGSS 2013 and 2015.

Dependent: Individual’s Income				
	Model 2	Model 3	Model 4	Model 5
Female ^b^ Male	0.167 *** (0.019)	0.151 *** (0.023)	0.137 *** (0.020)	0.137 *** (0.21)
FEdu1 ^b^ FEdu2		0.092 *** (0.012)	0.070 *** (0.016)	0.059 *** (0.010)
FEdu1 ^b^ FEdu3		0.126 *** (0.022)	0.129 *** (0.025)	0.127 *** (0.030)
FEdu1 ^b^ FEdu4		0.190 *** (0.024)	0.188 *** (0.023)	0.175 *** (0.026)
Health status	No	No	Positve	Positve
Social interaction	No	No	No	Positive
Control Group	Yes	Yes	Yes	Yes
Provincial effect	Yes	Yes	Yes	Yes
Year effect	Yes	Yes	Yes	Yes
Regional effect	Yes	Yes	Yes	Yes
R^2^	0.0711	0.0927	0.0981	0.0991
N	10,969	10,969	10,969	10,969

Robust standard errors are presented in parentheses. *** Denotes that the resulting coefficient is significant at the 1% level. ^b^ indicates the reference category.

**Table 8:** Interaction effect of gender, education, social interaction, and health on an individual’s income and employment CGSS 2013 and 2015.

Dependent Variables: Income and Employment						
	Model 1	Model 2	Model 3	Model 4	Model 5	Model 6
	Income	Emp	Income	Emp	Income	Emp
Main effect						
Female ^b^ Male	0.459 *** (0.027)	0.329 *** (0.047)				
hlth1 ^b^ Ghlth			0.326 *** (0.040)	0.320 *** (0.072)		
Hlth1 ^b^ Rhlth			0.486 *** (0.040)	0.419 *** (0.070)		
hlth1 ^b^ Vhlth			0.586 *** (0.046)	0.466 *** (0.081)		
Social1 ^b^ Social2					0.067 ** (0.032)	0.121 ** (0.056)
Social1 ^b^ Social3					0.060 * (0.034)	0.138 ** (0.059)
Two-way interaction effect						
Vhlth*Gender	−0.286 *** (0.084)	−0.778 *** (0156)				
Vhlth*Edu2			−0.530 *** (0.124)	0.429 ** (0.209)		
Vhlth*Edu3			−0.764 *** (0.133)	0.256 (0.232)		
Vhlth*Edu4			−0.851 *** (0.115)	−0.060 (0.198)		
Vhlth*Social2					−0.140 (0.097)	−0.053 (0.168)
Vhlth*Social3					0.029 (0.103)	−0.210 (0.177)
Control Group	Yes	Yes	Yes	Yes	Yes	Yes
Provincial effect	Yes	Yes	Yes	Yes	Yes	Yes
Year effect	Yes	Yes	Yes	Yes	Yes	Yes
Regional effect	Yes	Yes	Yes	Yes	Yes	Yes
R^2^/Pseudo R^2^	0.2365	0.1052	0.2258	0.1028	0.2128	0.1015
N	9164	9959	9149	9642	9163	9658

Robust standard errors are presented in parentheses. *** denotes that the resulting coefficient is significant at the 1% level. ** denotes that the resulting coefficient is significant at a 5% level. * Denotes that the resulting coefficient is significant at a 10% level. ^b^ indicates the reference category.

## Data Availability

General correspondence and requests for source data and materials should be addressed to Tajwar Ali. Requests for access to data should be addressed to tajooformanite@yahoo.com.
